# Supplementary material for: ATM-associated signalling triggers the unfolded protein response and cell death in response to stress
Source: Commun Biol. 2020 Jul 14;3:378. doi: 10.1038/s42003-020-1102-2 (PMC7360780; doi:10.1038/s42003-020-1102-2)
Supplement: Supplementary file 1 — Supplementary Information [file 42003_2020_1102_MOESM1_ESM.pdf]

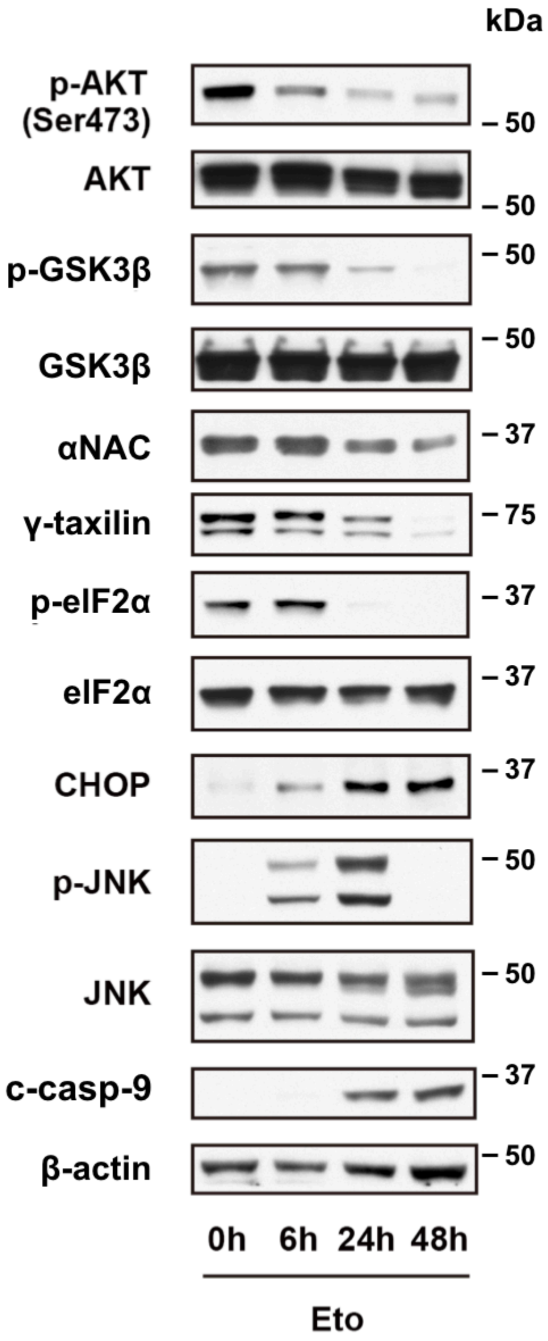

Supplementary Figure 1

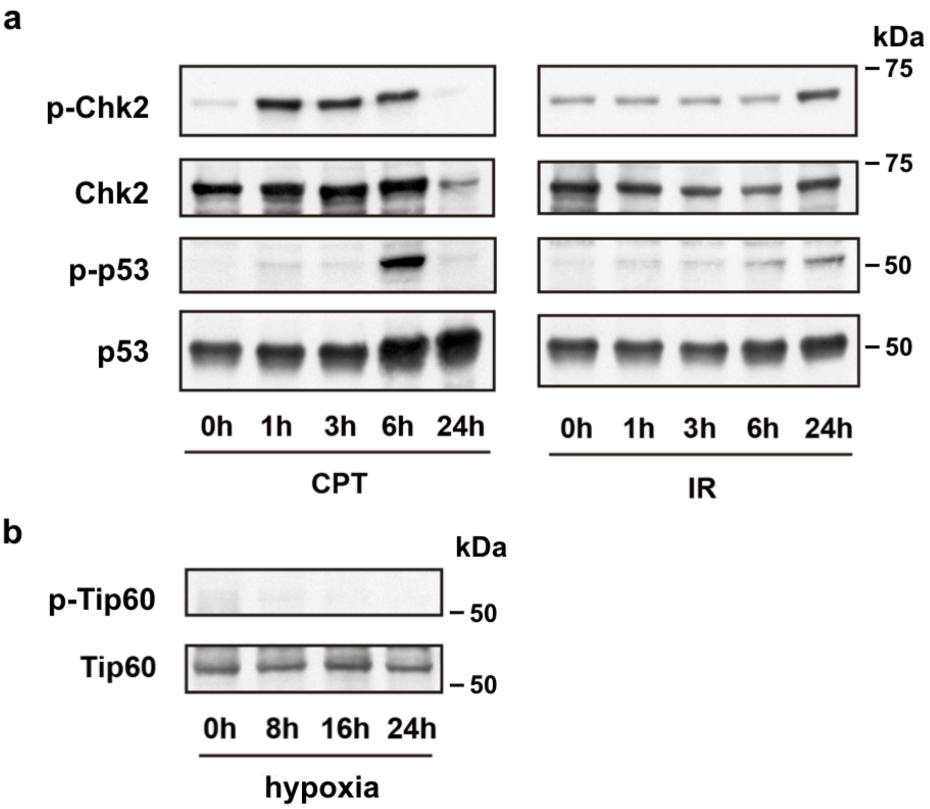

Supplementary Figure 2

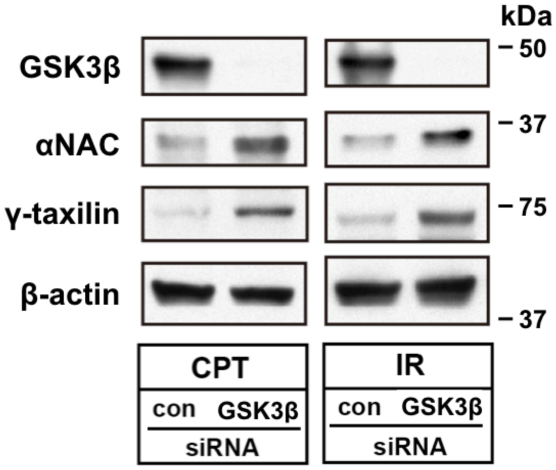

Supplementary Figure 3

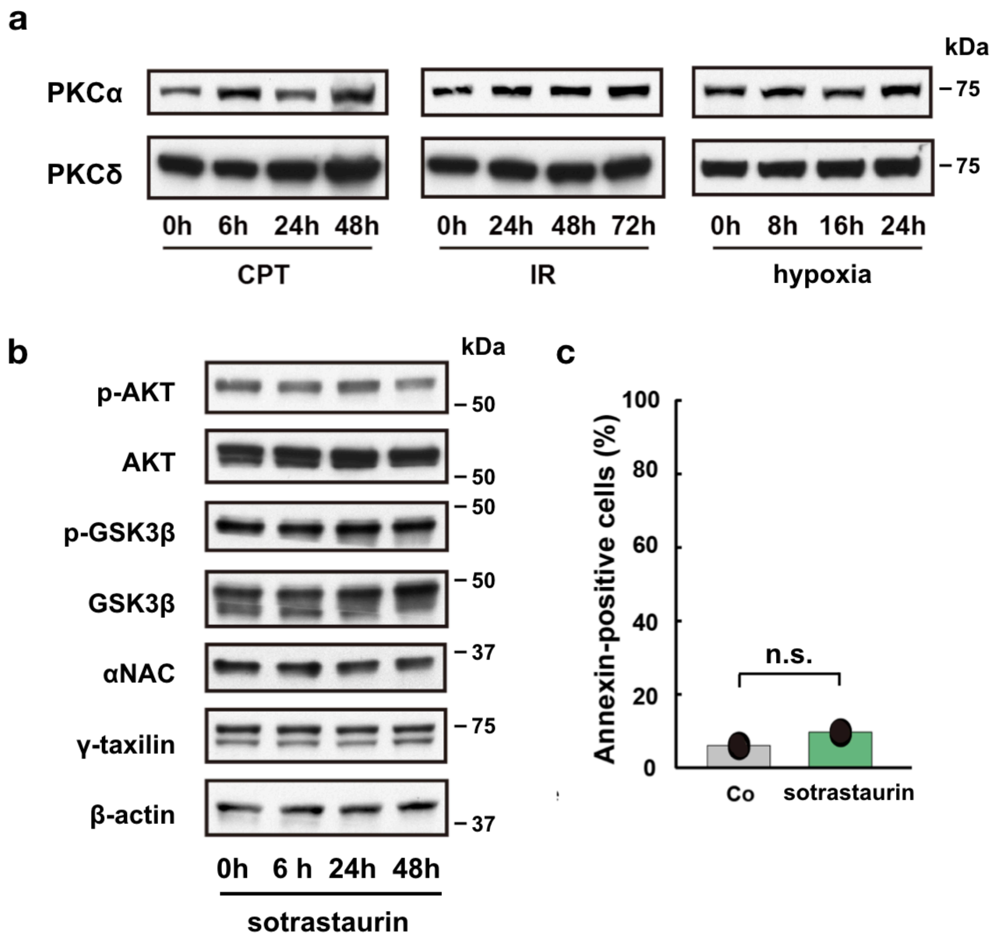

Supplementary Figure 4

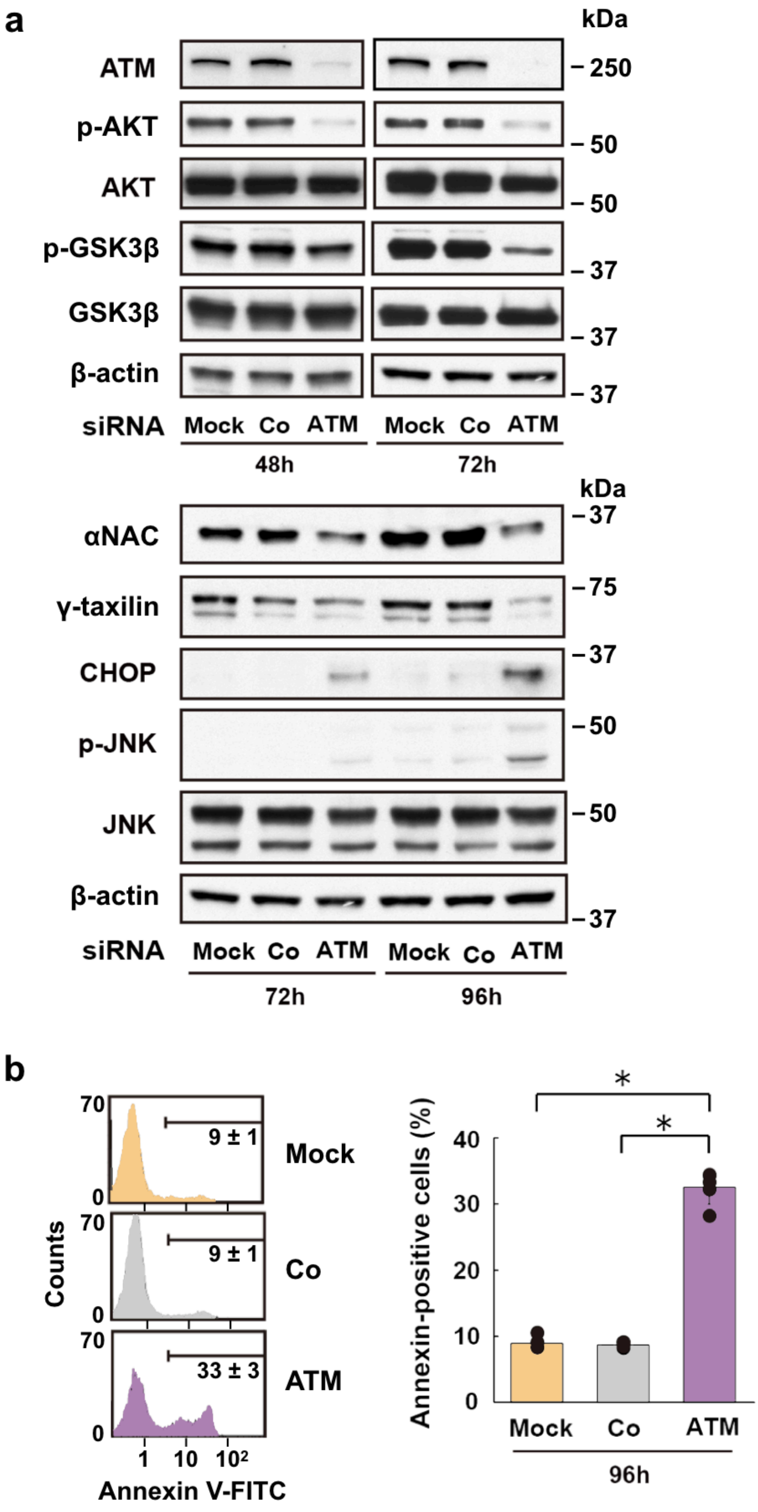

Supplementary Figure 5

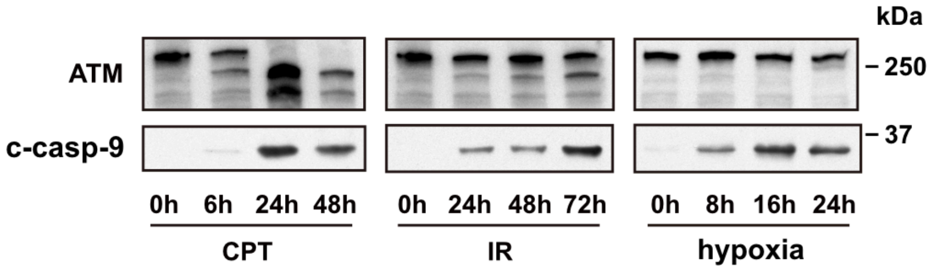

Supplementary Figure 6

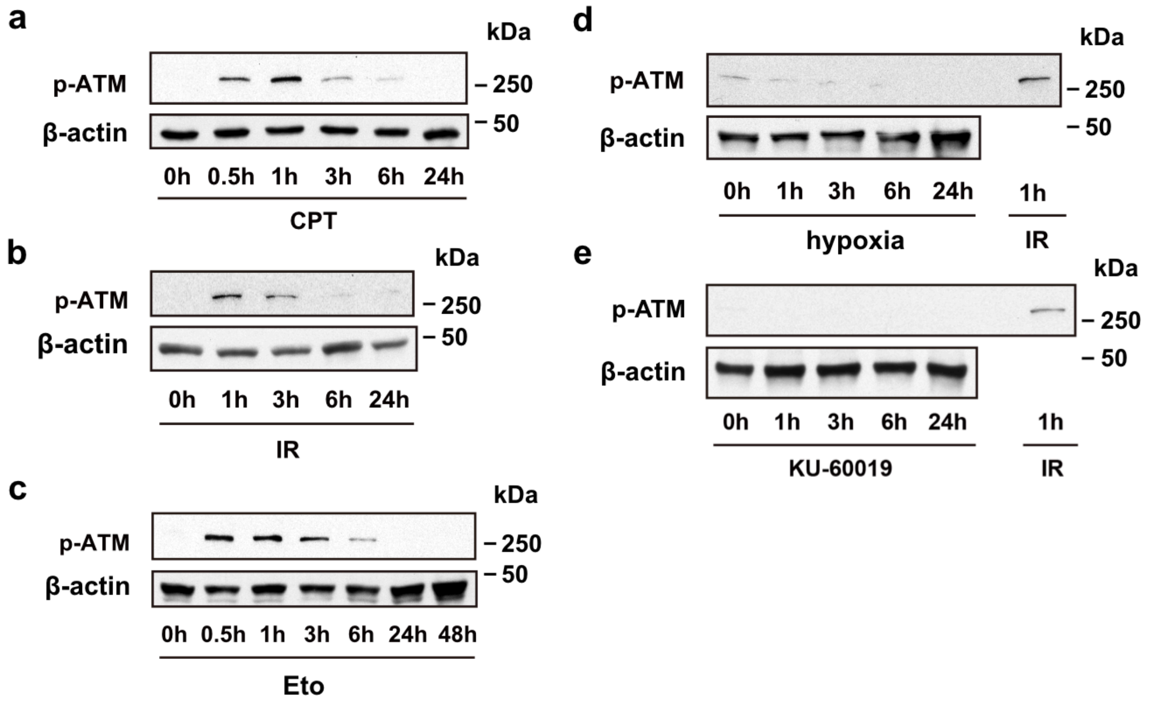

Supplementary Figure 7

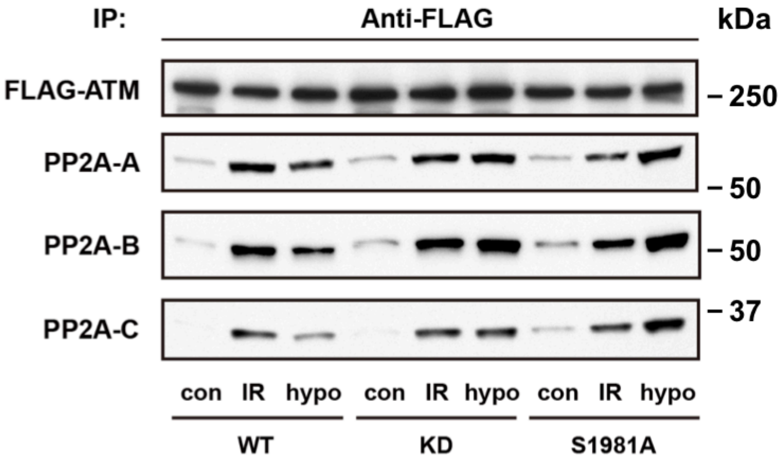

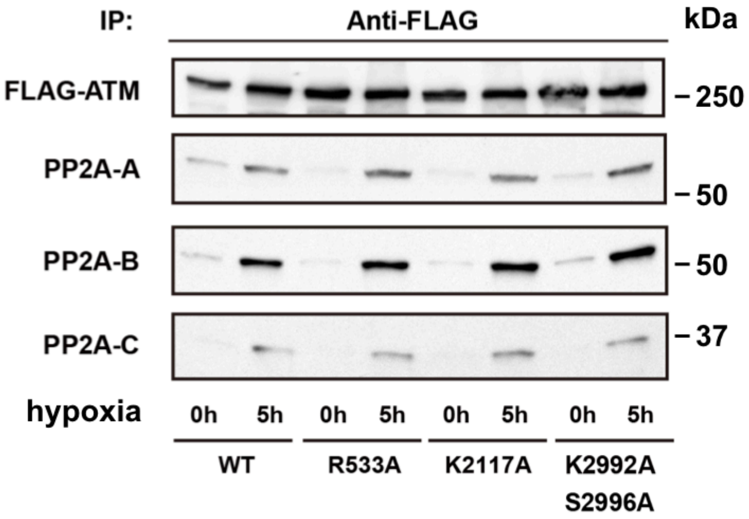

Supplementary Figure 8

Figure 1a

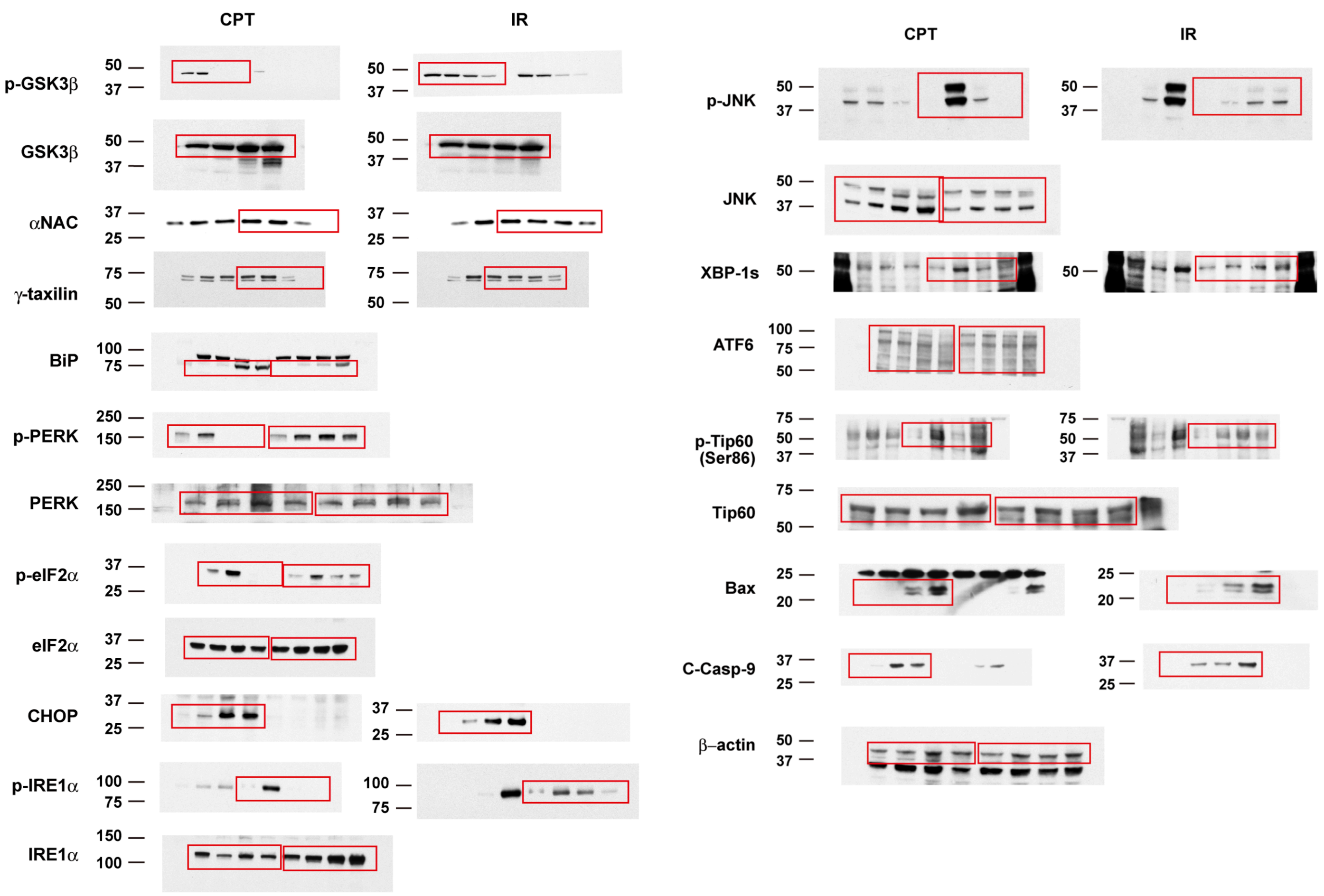

Figure 1d

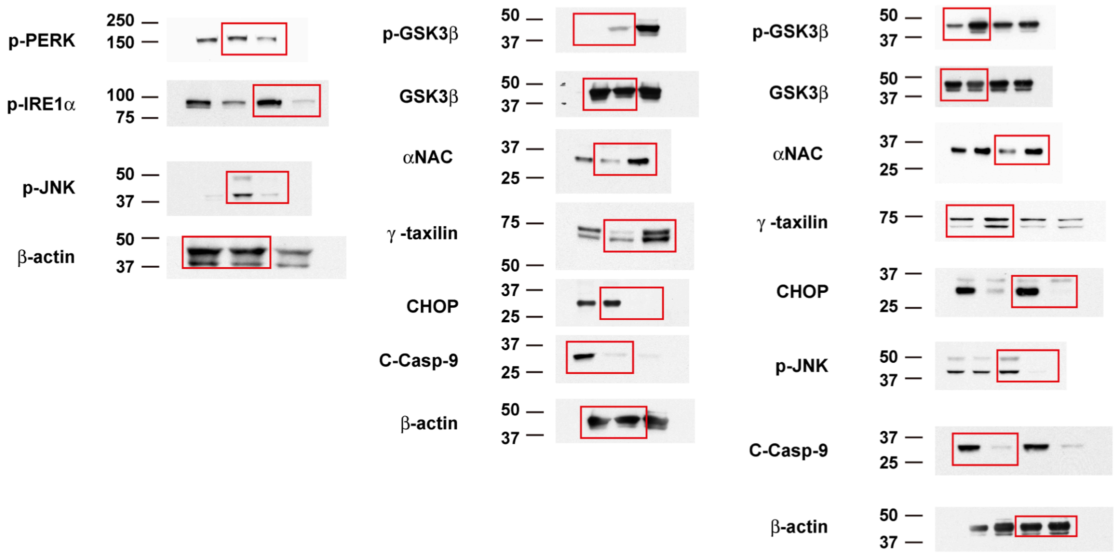

Supplementary Figure 9

Figure 2a

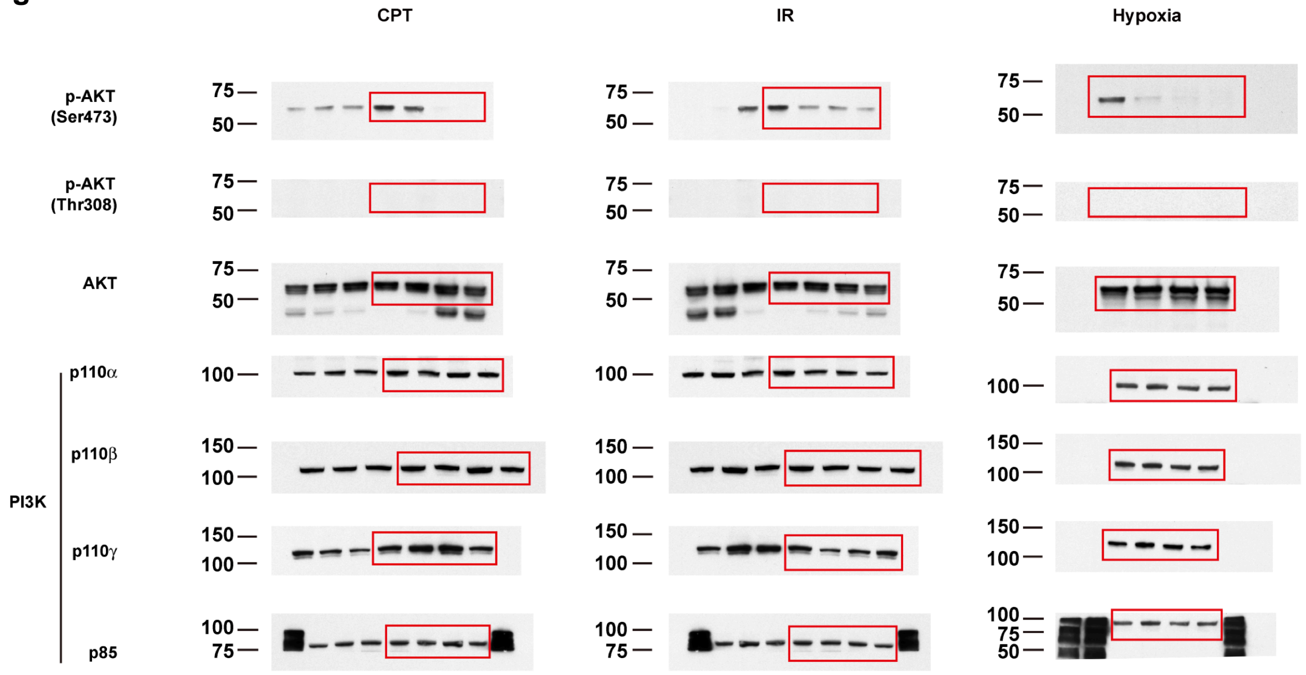

Figure 2b

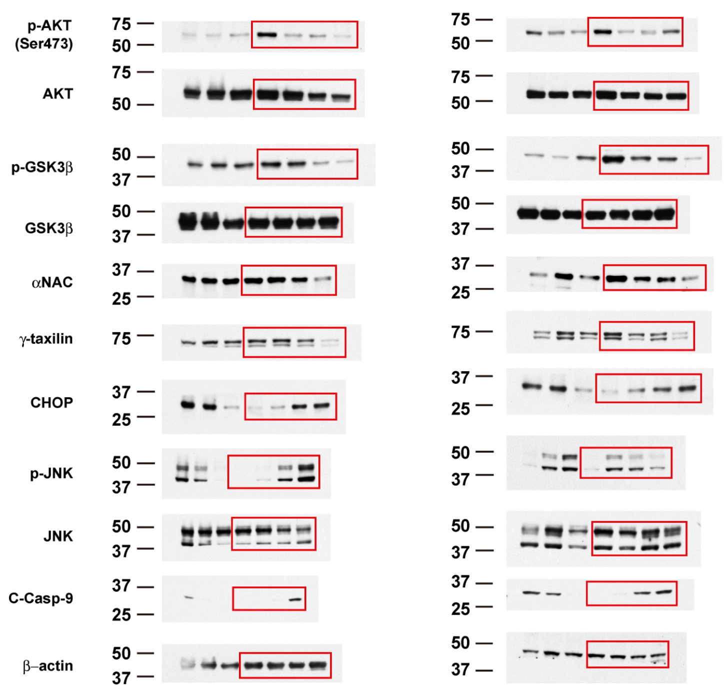

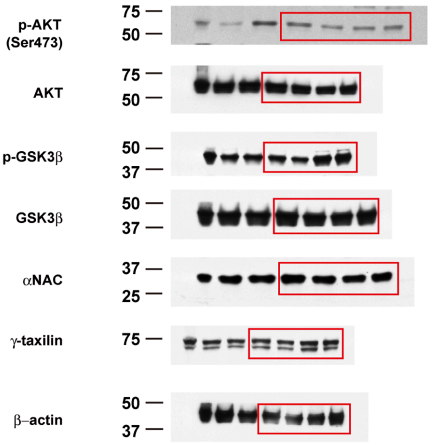

Figure 2d

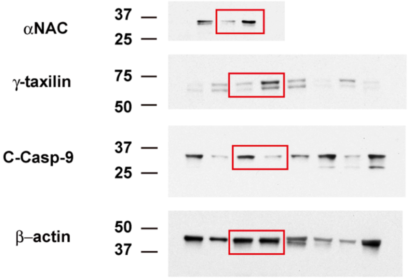

Figure 2f

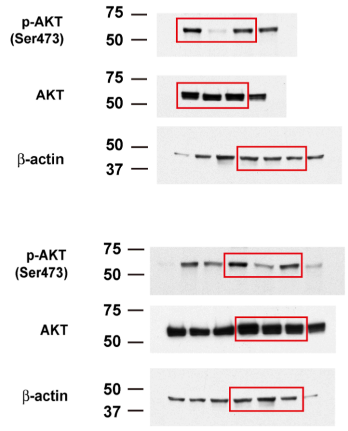

Figure 2g

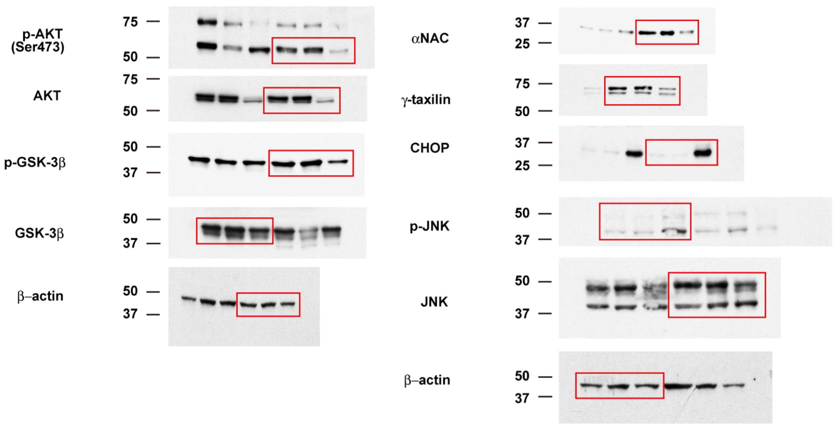

Supplementary Figure 9 (continued)

Figure 3b

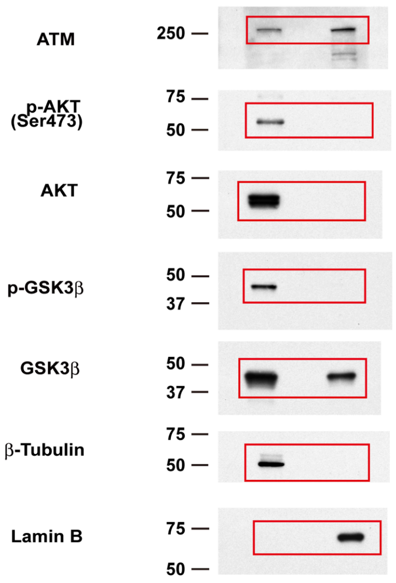

Figure 3e

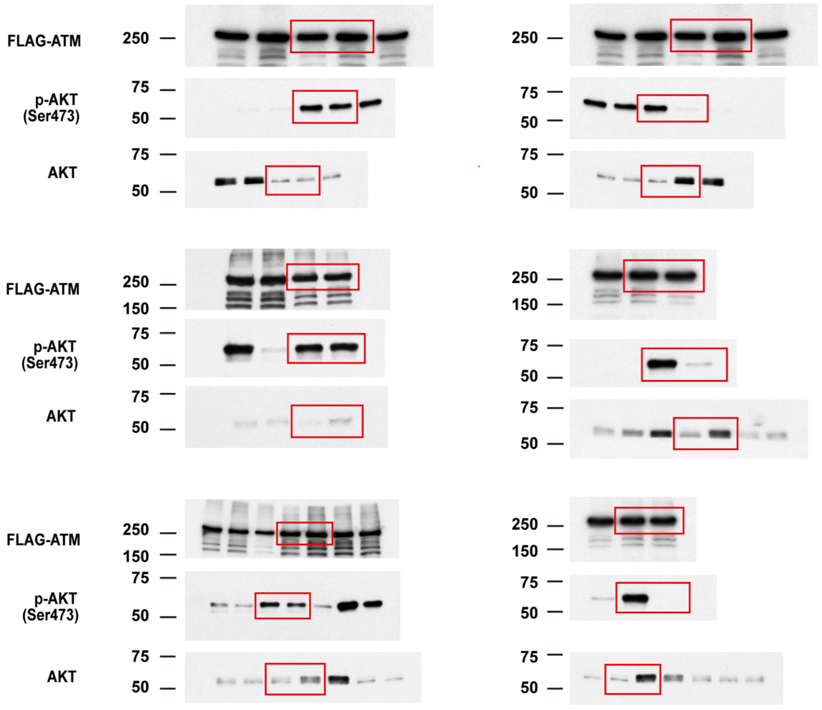

Figure 3d

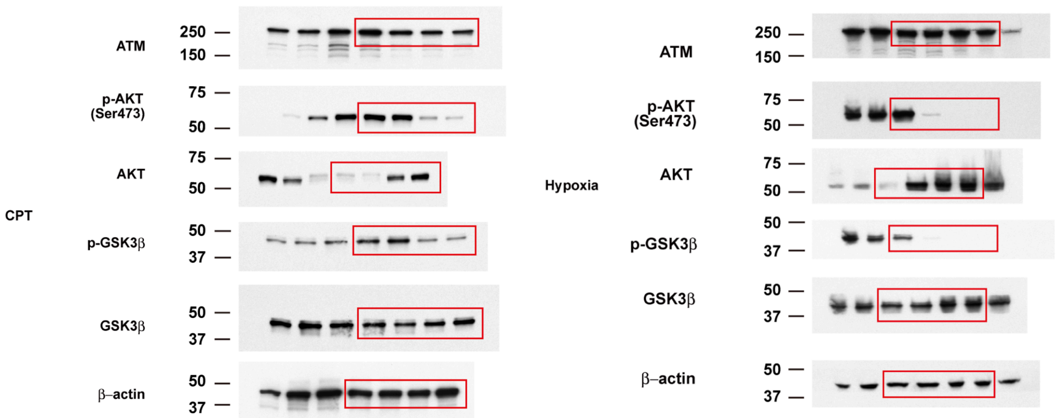

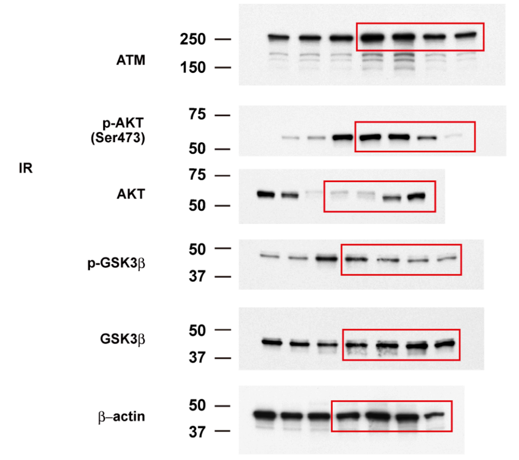

Figure 3g

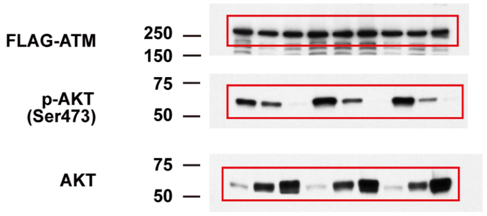

Figure 3h

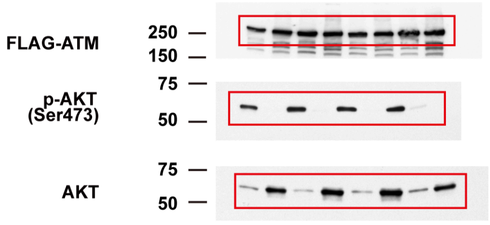

Figure 3f

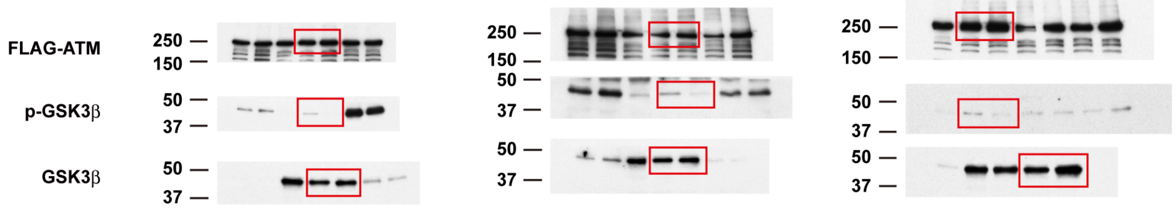

Supplementary Figure 9 (continued)

Figure 3i

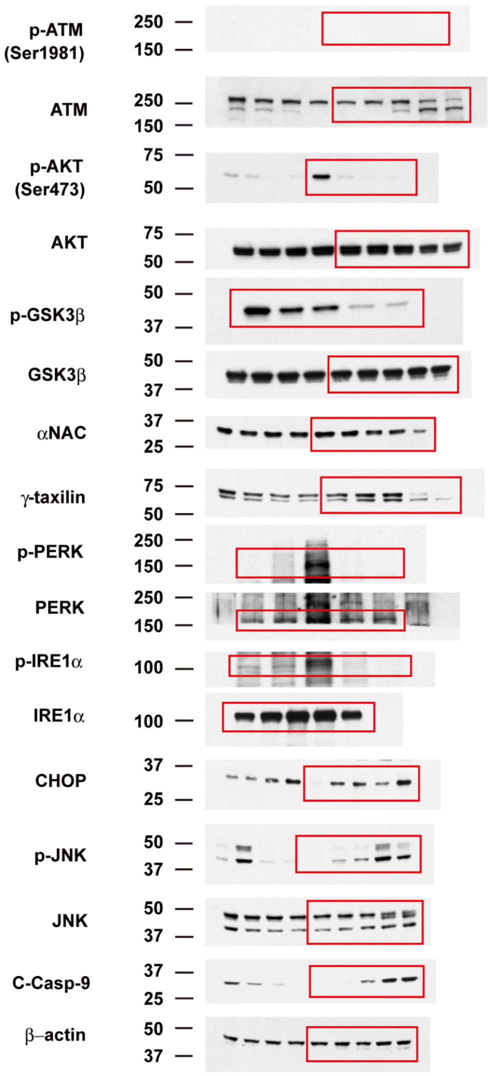

Figure 3k

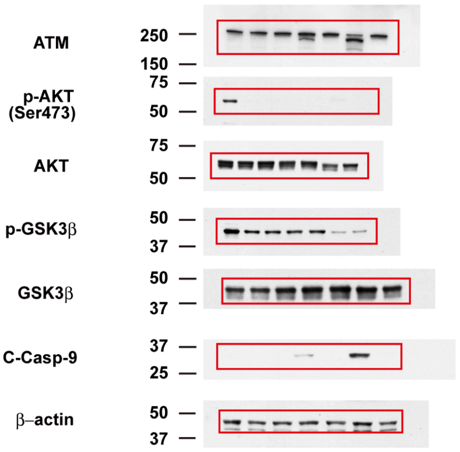

Supplementary Figure 9 (continued)

Figure 4a

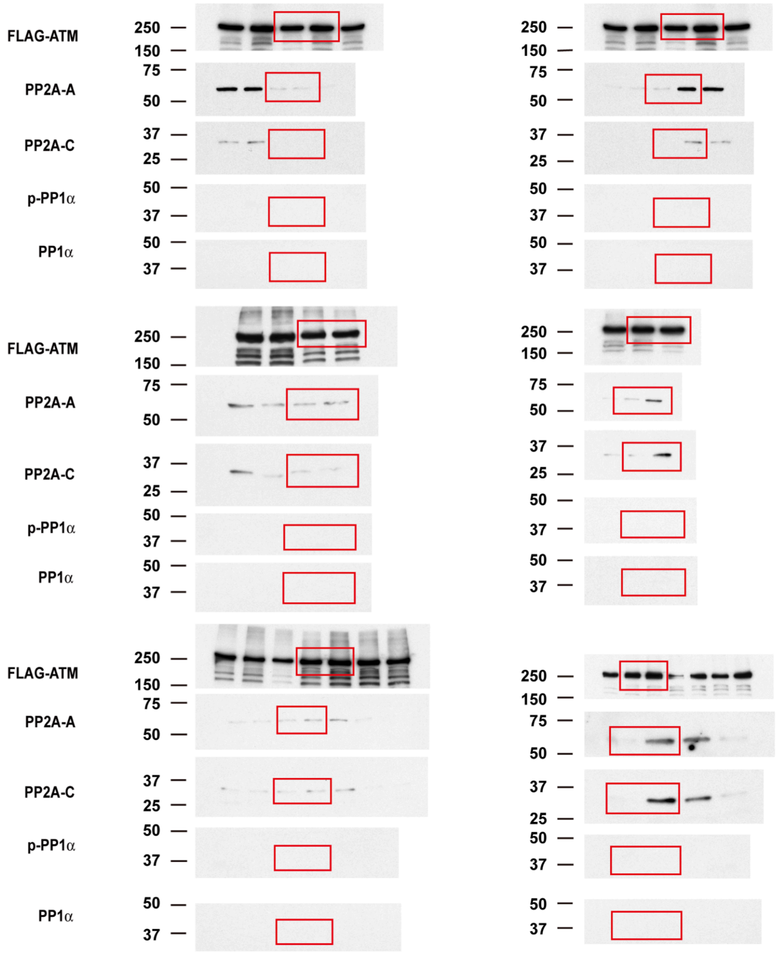

Figure 4b

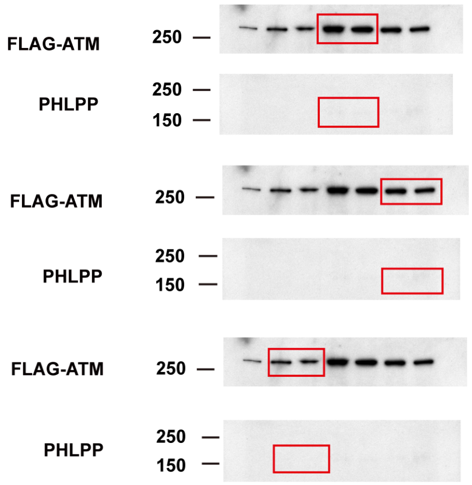

Figure 4c

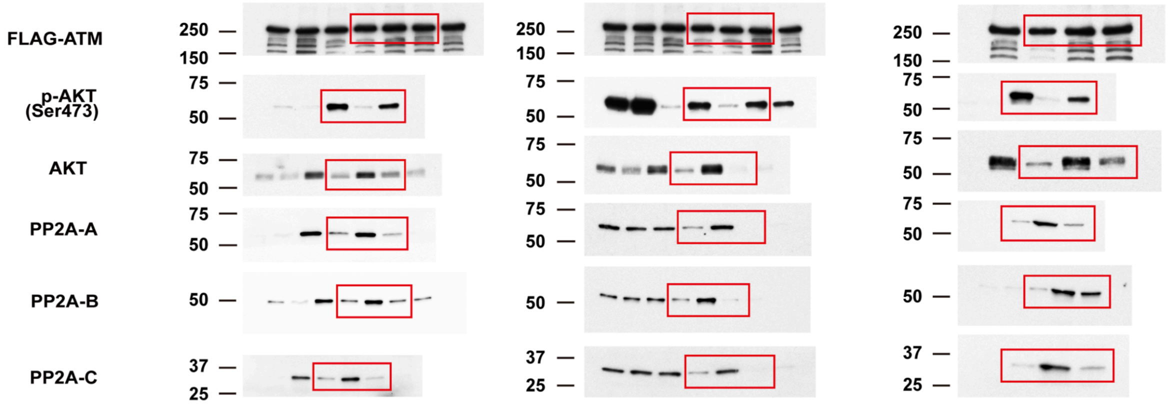

Figure 4f

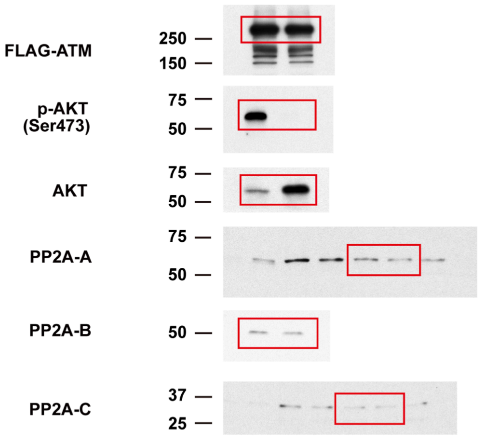

Figure 4g

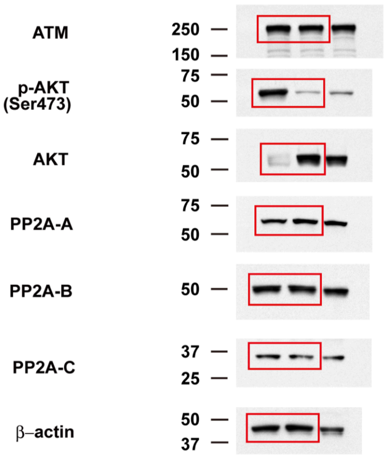

Supplementary Figure 9 (continued)

Supplementary Figure 1

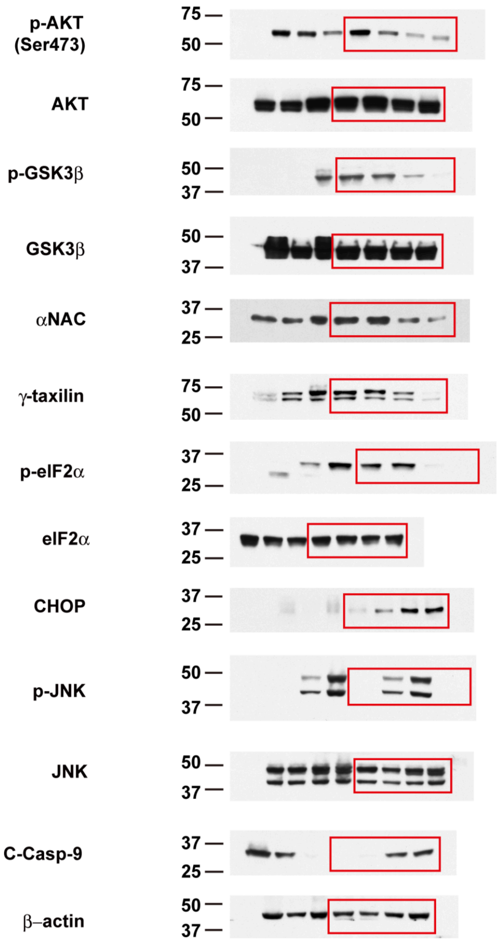

Supplementary Figure 3

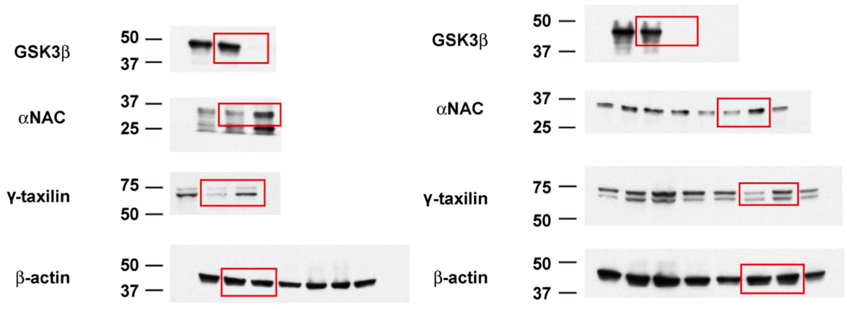

Supplementary Figure 4a

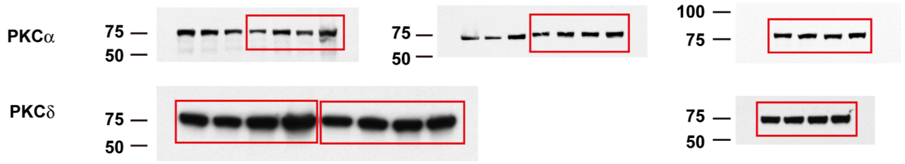

Supplementary Figure 4b

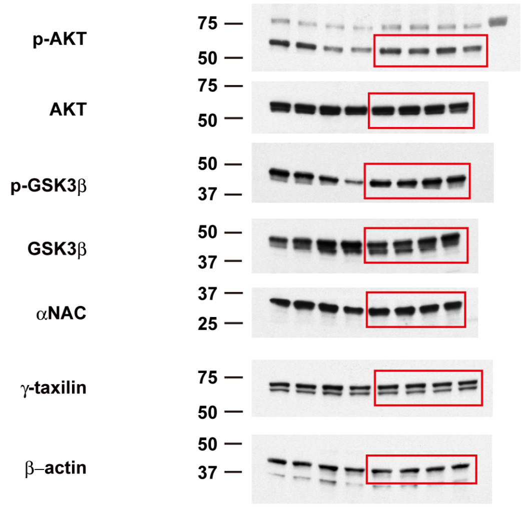

Supplementary Figure 2a

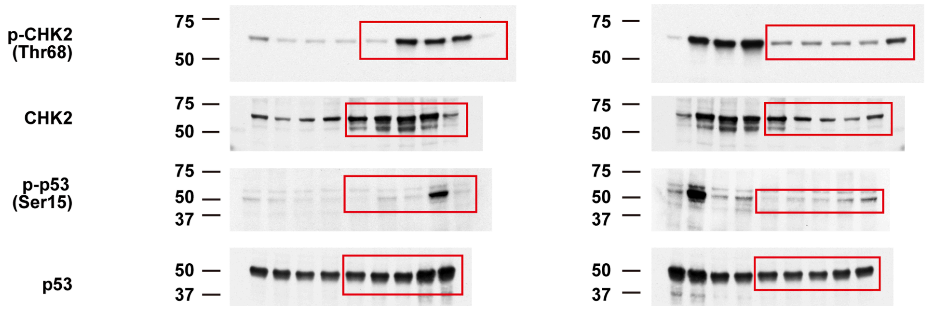

Supplementary Figure 2b

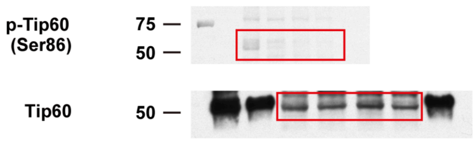

Supplementary Figure 9 (continued)

Supplementary Figure 5a

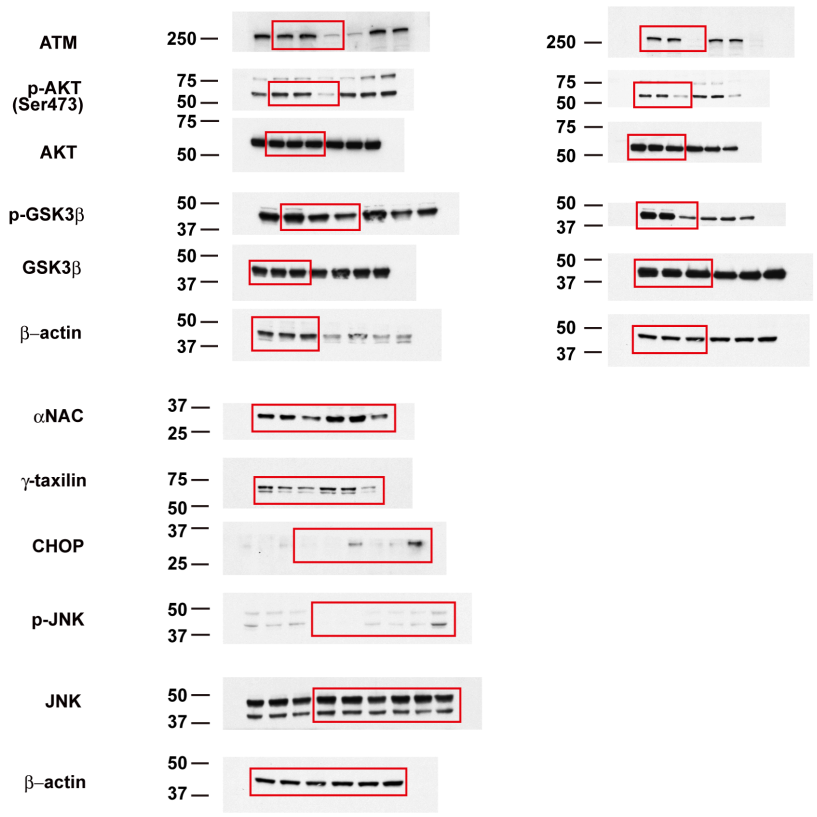

Supplementary Figure 8

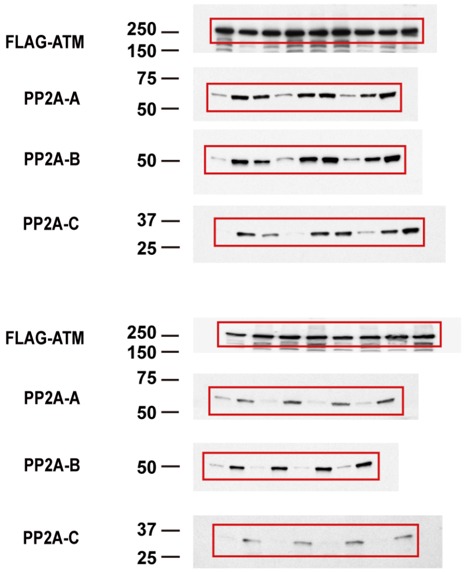

Supplementary Figure 6

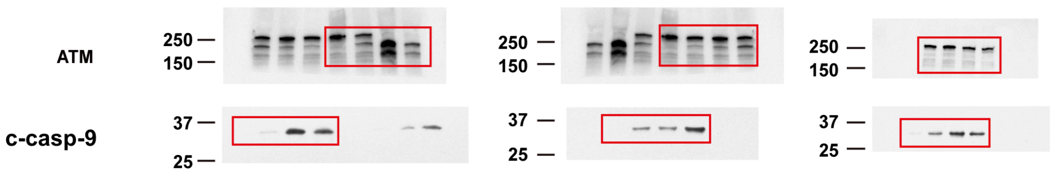

Supplementary Figure 7a

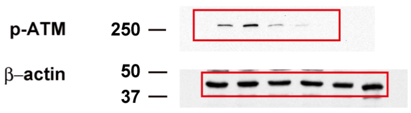

Supplementary Figure 7d

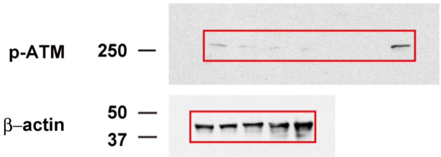

Supplementary Figure 7b

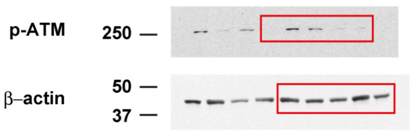

Supplementary Figure 7e

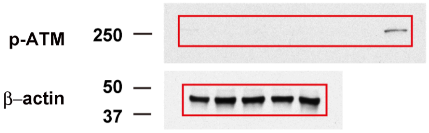

Supplementaty Figure 7c

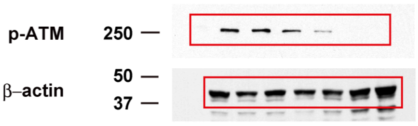

Supplementary Fig. 9 (continued)

Supplementary Table 1. Modification sites for FLAG-tagged ATM proteins in control, IR-treated, or hypoxic 293T cells

site	modification	best A-score	localization probability	the number of spectra for each modification					
				control		IR		hypoxic	
				modifited	total	modified	total	modified	total
S367	phosphorylation	60.18	1	1	7	0	3	0	4
K385	methylation	1000.00	1	8	18	6	15	5	12
K468	methylation	24.44	0.9964172	0	4	0	5	1	5
R533	methylation	1000.00	1	1	8	2	5	2	5
R568	methylation	1000.00	1	0	14	0	10	3	14
S1008	phosphorylation	44.18	0.9999235	2	20	0	18	0	18
K1398	methylation	1000.00	1	1	8	0	6	2	9
S1891	phosphorylation	34.46	0.9999932	2	10	1	12	1	7
S1981	phosphorylation	50.64	0.9999913	1	5	0	1	0	6
K2025	methylation	1000.00	1	0	7	0	7	1	12
K2117	methulation	1000.00	1	0	15	1	9	2	13
K2160	methylation	26.20	0.9976077	0	16	1	16	0	16
S2242	phsophorylation	1000.00	1	0	1	1	1	0	2
K2992	methylation	30.97	0.9999936	1	3	2	4	1	3
S2996	phosphorylation	138.75	1	1	4	0	0	0	1

Legends for Supplementary Figures

Supplementary Figure 1. AKT/GSK3β/αNAC/γTX and ER stress-related proteins in etoposide-treated HeLa S3 cells. Western blot analysis shows kinetic changes of p-AKT, AKT, p-GSK3β, GSK3β, αNAC, γTX, UPRs and apoptotic cell death-related proteins in HeLa S3 cells treated with etoposide (Eto, 10 μM). . Eto causes DNA strand breaks by interfering with topoisomerase II and has been shown to activate ER stress responses leading to caspase-dependent apoptotic cell death[1].

Supplementary Figure 2. Chk2, p53 and Tip60 protein expression in DNA-damaged (Chk2 and p53) or hypoxic (Tip60) HeLa S3 cells. a, Western blot analysis shows upregulation of p-Chk2 (Thr68) and p-p53 (Ser15) after CPT or IR treatment. **b**, Hypoxia does not induce Tip60 phosphorylation (Ser86), opposing to the results in the DNA-damaged cells (Fig. 1a).

Supplementary Figure 3. GSK3β depletion by RNA interference revives αNAC and γTX protein levels in cells under ER stress. Western blot analysis for αNAC and γ-taxilin in CPT- or IR-treated HeLa S3 cells with or without siRNA-mediated GSK3β depletion.

Supplementary Figure 4. PKC is not involved in ER stress-induced pro-apoptotic pathway. a, Western blot analysis for PKCα and PKCδ in CPT- or IR-treated, and hypoxic HeLa S3 cells. **b**, Western blot analysis for AKT, GSK3β, αNAC and γ-taxilin in sotrastaurin-treated HeLa S3 cells. **c**, FACS analysis for annexin-positive cell ratios after treatment with sotrastaurin. Bars, means ± s.e.m.; n = 6; n.s., not statistically significant, t-test.

Supplementary Figure 5. ATM ablation induces ER stress responses. a, Western blot analysis shows kinetic changes in expression levels of αNAC, γTX, AKT, p-AKT, GSK3β and p-GSK3β, and activation of death signals (CHOP and p-JNK) in HeLa S3 cells that are depleted of ATM by RNA interference. Protein expression profiles in the ATM-depleted cells were reminiscent of those treated with the other ER stress induces tested (Fig. 1a) or the pharmacological ATM

inhibitor KU-60019 (Fig. 3i) **b**, FACS analysis shows enhanced apoptotic cells death after ATM RNA interference. Bar graph shows increased annexin-positive cell ratios in ATM-depleted cells 96 h after the siRNA addition in comparison with control or mock-treated cells. The ATM ablation was less effective for apoptosis induction compared with the other ER stress induces tested (Fig. 1b) and KU-60019 (Fig. 3j). Bars, mean ± s.e.m; n = 5; * p <0.001, Tukey-Kramer test.

Supplementary Figure 6. ATM protein degradation under ER stress. Western blot analysis shows CPT- or IR-induced degradation of ATM protein and caspase-9 activation in HeLa S3 cells (left and middle panels). Hypoxic treatment also activated caspase-9 but ATM degradation was not obvious compared with CPT or IR treatment, without degraded proteins having lower molecular weights (right panel).

Supplementary Figure 7. ATM phosphorylation kinetics after DNA damage, hypoxic stress, or ATM inhibition. a-e, Western blot analysis shows kinetic changes in p-ATM protein expression levels after DNA damage with CPT (1 μM, **a**), IR (20 Gy, **b**), or Eto (10 μM, **c**); or under hypoxia (**d**), or following ATM inhibition by KU-60019 (20 μM, **e**). As expected, ATM was transiently phosphorylated at Ser1981 within the first 0.5 - 1 h after CPT, IR, or Eto treatment (**a-c**). In contrast, hypoxic stress or KU-60019 did not induce ATM phosphorylation. ATM phosphorylation was reportedly upregulated in GM0536 human lymphoblastic cells 3-6 h after the start of cultivation under hypoxic conditions[2]; however, this was not the case with the present study with HeLa S3 cells. These results suggest that ATM phosphorylation may not directly contribute to the AKT activation mechanisms in ER stress-induced cell death.

Supplementary Figure 8. Effects of ATM mutations on the interaction with PP2A phosphatase. Immunoprecipitation assay for PP2A phosphatase (PP2A-A, PP2A-B and PP2A-C) in the FLAG-tagged wild-type (WT) or mutated (kinase-dead [KD] or S1981A in upper panel; or R533A, K2117A, or K2992A/S2996A in lower panel) ATM precipitates from IR-treated or hypoxic (hypo) 293T cells.

Supplementary Figure 9. Uncropped blots for Figures 1-4 and Supplementary Figures 1-8

Enclosed blot areas are used for corresponding figure parts.

Source Data 1. Data list shows source data underlying the graphs presented in the main Figures (Figure 1b, e-h; Figure 2c, e, h; Figure 3j; and Figure 4d, e).

References for Supplementary Figures

1. Wang, C., et al. Etoposide induces apoptosis in activated human hepatic stellate cells via ER stress. *Sci. Rep.* **6**, 34330 (2016).

2. Bencokova, Z., et al. ATM activation and signaling under hypoxic conditions. *Mol. Cell Biol.* **29**, 526-537 (2009).
