# Supplementary material for: ATM-associated signalling triggers the unfolded protein response and cell death in response to stress
Source: Commun Biol. 2020 Jul 14;3:378. doi: 10.1038/s42003-020-1102-2 (PMC7360780; doi:10.1038/s42003-020-1102-2)
Supplement: Supplementary file 4 — Reporting Summary [file 42003_2020_1102_MOESM4_ESM.pdf]

nature research

Corresponding author(s): Takashi Nakamura

Last updated by author(s): Jun 13, 2020

Reporting Summary

Nature Research wishes to improve the reproducibility of the work that we publish. This form provides structure for consistency and transparency in reporting. For further information on Nature Research policies, see our Editorial Policies and the Editorial Policy Checklist.

Statistics

For all statistical analyses, confirm that the following items are present in the figure legend, table legend, main text, or Methods section.

n/a	Confirmed	
☐	☒	The exact sample size (*n*) for each experimental group/condition, given as a discrete number and unit of measurement
☐	☒	A statement on whether measurements were taken from distinct samples or whether the same sample was measured repeatedly
☐	☒	The statistical test(s) used AND whether they are one- or two-sided<br>*Only common tests should be described solely by name; describe more complex techniques in the Methods section.*
☒	☐	A description of all covariates tested
☐	☒	A description of any assumptions or corrections, such as tests of normality and adjustment for multiple comparisons
☐	☒	A full description of the statistical parameters including central tendency (e.g. means) or other basic estimates (e.g. regression coefficient) AND variation (e.g. standard deviation) or associated estimates of uncertainty (e.g. confidence intervals)
☐	☒	For null hypothesis testing, the test statistic (e.g. *F*, *t*, *r*) with confidence intervals, effect sizes, degrees of freedom and *P* value noted<br>*Give P values as exact values whenever suitable.*
☒	☐	For Bayesian analysis, information on the choice of priors and Markov chain Monte Carlo settings
☒	☐	For hierarchical and complex designs, identification of the appropriate level for tests and full reporting of outcomes
☒	☐	Estimates of effect sizes (e.g. Cohen's *d*, Pearson's *r*), indicating how they were calculated

Our web collection on statistics for biologists contains articles on many of the points above.

Software and code

Policy information about availability of computer code

Data collection	Mass spectroscopy: Waters NanoAcquity interfaced to Thermo-Fisher Q Extractive; Microscopy: TCS SP2 confocal microscope (Leica); Flow cytometry: Epics XL (Beckmann Coulter); Microplate reader: Wallac 1420
Data analysis	Data analysis for spectroscopy was performed using Byonic and Byologic (Protein Metrics). All statistical analyses were performed using SPSS (IBM).

For manuscripts utilizing custom algorithms or software that are central to the research but not yet described in published literature, software must be made available to editors and reviewers. We strongly encourage code deposition in a community repository (e.g. GitHub). See the Nature Research guidelines for submitting code & software for further information.

Data

Policy information about availability of data

All manuscripts must include a data availability statement. This statement should provide the following information, where applicable:

- Accession codes, unique identifiers, or web links for publicly available datasets
- A list of figures that have associated raw data
- A description of any restrictions on data availability

All data including a list of figures that have associated raw data and mass spectroscopy are available in main text (Figures 1-4) and Supplementary Figures 1-9, Supplementary Table 1, and Supplementary Data.

Field-specific reporting

Please select the one below that is the best fit for your research. If you are not sure, read the appropriate sections before making your selection.

- [x] Life sciences
- [ ] Behavioural & social sciences
- [ ] Ecological, evolutionary & environmental sciences

For a reference copy of the document with all sections, see nature.com/documents/nr-reporting-summary-flat.pdf

Life sciences study design

All studies must disclose on these points even when the disclosure is negative.

Sample size	All the experiments were performed using cell culture and no power calculation was performed to determin the sample size. However, the sample size was chosen based on our previous experiments and publications (references 1 and 2, PMID: 19609276, PMID: 25880086). Statistically significant (p <0.001) differences were typically obtaiined with repeated (n = 3-6) and independent experiments with relatively small s.e.m.
Data exclusions	No data was excluded from the analysis.
Replication	Each series of FACS experiments were performed in triplicate or more (n = 3-6). All attempts at replication were successful with relatively small s.e.m. Independent and repeated experiments were also performed for Western blot and immunoprecipitation analyses, and representative data from consistent results were presented in figures.
Randomization	All the experiments were performed by two researchers, with each performing separate experiments in this study. Therefore, we did not randomized the experiments.
Blinding	Blind analysis was performed only in confocal microscopic analyses, i.e., immunostaining and proximity ligation assay (PLA), where cell culture/treatment and imaging were performed separately by two different researchers in a blind fashion, and the results were analyzed and interpreted also in a blind fashion by a third researcher (= corresponding author).

Reporting for specific materials, systems and methods

We require information from authors about some types of materials, experimental systems and methods used in many studies. Here, indicate whether each material, system or method listed is relevant to your study. If you are not sure if a list item applies to your research, read the appropriate section before selecting a response.

Materials & experimental systems

n/a	Involved in the study	
☐	☒	Antibodies
☐	☒	Eukaryotic cell lines
☒	☐	Palaeontology and archaeology
☒	☐	Animals and other organisms
☒	☐	Human research participants
☒	☐	Clinical data
☒	☐	Dual use research of concern

Methods

n/a	Involved in the study	
☒	☐	ChIP-seq
☐	☒	Flow cytometry
☒	☐	MRI-based neuroimaging

Antibodies

Antibodies used	The primary antibodies used for Western blotting included: γ-taxilin (Santa Cruz Biotechnology, sc-393610, used at a concentration of 1:1000), αNAC (Abnova, H00342538, 1:1000), ATF6 (IMGENEX, IMG-273, 1:200), BiP (KDEL; Stressgen, spA-827, 1:200), CHOP (Cell Signaling, 2895, 1: 1000), PERK (Santa Cruz Biotechnology, sc-13073, 1:200), phospho-PERK (Santa Cruz Biotechnology, sc-32577, 1:200), IRE1α (Cell Signaling, 3294, 1:1000), phospho-IRE1α (NOVUS, NB100-2323, 1:1000; abcam, ab124945, 1:1000), cleaved caspase-9 (Cell Signaling, 9505, 1:500), eIF2α (Cell Signaling, 9722, 1:1000), phospho-eIF2α (Cell Signaling, 9721, 1:500), GSK3β (Cell Signaling, 9315, 1:2000), phospho-GSK3β (Ser9, Cell Signaling, 9336, 1:1000), JNK (Cell Signaling, 9252, 1:1000), phospho-JNK (Cell Signaling, 4668, 1:500), Bax (Santa Cruz Biotechnology, sc-748, 1:200), p53 (Merck Millipore, Ab-6, 1:1000), phospho-p53 (Ser15, DNA damage antibody sampler kit, Cell Signaling, 9947, 1:500), Chk2 (Cell Signaling, 2662, 1:2000), phospho-Chk2 (DNA damage antibody sampler kit; Cell Signaling, 9947, 1:1000), Tip60 (Cell Signaling, 12058, 1:1000), phospho-Tip60 (Ser86, Abcam, ab73207, 1:500), XBP1s (Santa Cruz Biotechnology, sc-7160, 1:200), PI3K (Cell Signaling, p110α, 4249, 1:1000; p110β, 3011, 1:1000; p110γ, 5405, 1:1000; p85, 4257, 1:1000), ATM (Cell Signaling, 2873, 1:2000), phospho-ATM (Ser1981; Cell Signaling, 5883, 1:500), AKT (Cell Signaling, 4691, 1:2000), phospho-AKT (Ser473, Cell Signaling, 4060, 1:500), phosphor-AKT (Ser308, Cell Signaling, 13038, 1:500), PKCα (Cell Signaling, 2056, 1:1000), PKCδ (Cell Signaling, 9616, 1:1000), PP2A-A (Cell Signaling, 2041, 1:1000), PP2A-B (Cell Signaling, 2290, 1:1000), PP2A-C (Cell Signaling, 2259, 1:1000), PP1α (Cell Signaling, 2582, 1:500), phospho-PP1α (Thr320; Cell Signaling, 2581, 1:500), PHLPP (Bethyl, A300-661A, 1:1000), FLAG (Sigma, F7425, 1:1000), β-tubulin (Cell Signaling, 2146, 1:1000), lamin B (Cell Signaling, 12586, 1:1000), β-actin (Sigma, A3853, 1:1000). The secondary antibodies used for Western blotting included anti-rabbit (Cell Signaling, 7074, 1:1000) and anti-mouse (Cell Signaling, 7076, 1:1000) IgG antibodies. The primary antibodies used for

immunofluorescence microscopy included ATM (Merck Millipore, 07-1286, 1:100), p-AKT (Santa Cruz Biotechnology, sc-514032, 1:100) and phospho-GSK3β (Thermo Fisher, MA5-14873, 1:100; Santa Cruz Biotechnology, 373800, 1:100). The secondary antibodies used for immunofluorescence microscopy included FITC (Invitrogen, F2765, 1:400) and Cy3 (GE Healthcare, PA 43009, 1:2500).

Validation

The validation of the antibodies used in this study was performed based on the statements and reference data on ththe e manufacture's website. However, some antibodies (i.e., p-IRE1α, abcam ab124945 and novus NB100-2323 for Western blotting; and p-GSK3β, Santa Cruz Biotechnology 373800 and Thermo-Fisher MA5-14873 for immunoprecipitation assay) were validated by using two products from different manufactures and comparing the results.

Eukaryotic cell lines

Policy information about cell lines

Cell line source(s)

We used HeLa S3 human cervical cancer cells and 293T human embryonic kidney cells, which were both obtained from ATCC.

Authentication

None of the cell lines used were authenticated.

Mycoplasma contamination

The cell lines were not tested for mycoplasma contamination.

Commonly misidentified lines
(See ICLAC register)

The cell lines used in this study are not listed as commonly misidentified lines (ICLAC).

Flow Cytometry

Plots

Confirm that:

- [x] The axis labels state the marker and fluorochrome used (e.g. CD4-FITC).
- [x] The axis scales are clearly visible. Include numbers along axes only for bottom left plot of group (a 'group' is an analysis of identical markers).
- [x] All plots are contour plots with outliers or pseudocolor plots.
- [x] A numerical value for number of cells or percentage (with statistics) is provided.

Methodology

Sample preparation

Apoptosis was assessed after incubating with Annexin V-FITC (Sigma) at room temperature for 10 min, then cells positive for annexin-V were analyzed by FACS scan.

Instrument

Samples were sorted on an Epics XL (Beckmann Coulter).

Software

Flow cytometry data were analyzed using the software that is pre-installed in the instrument used.

Cell population abundance

Each experiment group contained 10000 cells. Due to the relatively low cell population, the abundance of the population in the post-sort fractions was not assessed.

Gating strategy

Apoptotic cells were identified as annexin-V-positive cells.

- [x] Tick this box to confirm that a figure exemplifying the gating strategy is provided in the Supplementary Information.
